# Mass loss, timing and duration of catastrophic moult in little penguins

**DOI:** 10.1242/bio.061989

**Published:** 2025-09-02

**Authors:** Naomi C. A. Wells, Maddison J. Ledwidge, Peter Dann, Melissa J. Walker, John P. Y. Arnould

**Affiliations:** ^1^ School of Life and Environmental Sciences, Faculty of Science, Engineering and Built Environment, Deakin University, Burwood VIC 3125, Australia; ^2^ Bellarine Catchment Network, Drysdale, VIC 3222, Australia; ^3^ Conservation Department, Phillip Island Nature Parks, Cowes, Phillip Island, VIC 3991, Australia

**Keywords:** Integument, Feather, Phillip Island, Prey availability, Environmental conditions

## Abstract

Feather regeneration is vital for birds’ thermoregulation, courtship, breeding, camouflage, and locomotion, with strategies reflecting life history. Little penguins (*Eudyptula minor*) undergo catastrophic moult, replacing all feathers within a short timeframe while on land and not foraging. This study examined the 2015 and 2016 moult seasons on Phillip Island to explore factors influencing moult timing, duration, and mass. Moult started 9.6 days earlier in 2016 (∼Feb. 15) than in 2015 (∼Feb. 24), and year was found to be the only significant predictor of this moult start date. Moult duration was similar between years (medians: 18.0 days in 2015, 17.5 in 2016) and only slightly reduced with later start dates (−0.04 days per day delay; ∼58 min). Average daily mass loss during moult were best explained by moult duration and starting mass, with longer moult and greater starting mass leading to greater mass loss. The timing and duration of little penguins’ moult, along with the need for significant pre-moult mass gain, are likely influenced by external factors like local prey availability. Moult plasticity likely benefits little penguin survival.

## Teaser

**Summary:** This study explores factors affecting little penguin (*Eudyptula minor*) moult timing, duration, and mass loss.

## INTRODUCTION

Birds and mammals maintain a protective integument, composed of an individual's dermal skin layer as well as specialised extensions of the epidermis such as hair, whiskers and feathers that degrade over time (Higgins, 2014; Alesci et al., 2023). Some species overcome degradation by replacing this integument through a process called ‘moult’ that can alter the size, structure, or appearance of the integument (Dixit and Sougrakpam, 2013; Lees et al., 2014; Guallar et al., 2014). Feathers, one of the most specialised forms of integument, serve in functions such as flight, thermoregulation, camouflage, waterproofing and sexual or territorial displays. While feather synthesis requires substantial energy resources (Cornelius et al., 2011), complete replacement of a bird's plumage is a fundamental requirement of their long-term survival and success (Rogers et al., 2014; Terrill and Shultz, 2023). Penguins (family Spheniscidae) are a group of flightless seabirds found primarily in the southern hemisphere (Pelegrín and Acosta Hospitaleche, 2022), and are highly adapted to the marine environment, having a full body plumage composed of thousands of pennaceous feathers that interlock to form a tight, insulated waterproof barrier (Clarke et al., 2010). A penguin's plumage needs to be well maintained as they rely upon it within all aspects of the annual cycle (Gauthier-Clerc et al., 2002; Enstipp et al., 2019). In contrast to most birds that progressively replace their feathers throughout the year, penguins exhibit a unique strategy called ‘catastrophic moult’, whereby the entire plumage is replaced in one concentrated timeframe (Carpenter-Kling et al., 2022).

Penguins complete catastrophic moult ashore as the loss of feathers compromises the waterproof nature of the plumage, which would usually provide slipstream required for efficient swimming to catch prey, and insulation against cold sea temperatures (Ganendran et al., 2016; Williams et al., 2015). Given these impacts, penguins must fast throughout the entire moult process (Beltran et al., 2018; Lewden et al., 2024), which can result in the loss of up to 58% of pre-moult body mass at the completion of moult due to the cost of feather synthesis and increased thermoregulatory demands as a result of feather loss (Enstipp et al., 2019; Whitehead et al., 2017). To survive this fast, penguins will double their body mass by accumulating endogenous protein and lipid reserves prior to moult in preparation for this fast which, depending on the species, can take up to 2-5 weeks (Enstipp et al., 2019). Once the moult is complete, penguins immediately return to sea to forage and reestablish body condition (Carpenter-Kling et al., 2022). As the energetic cost of fasting is amplified by heightened thermoregulatory demand and moult cannot be stopped once it has commenced, a penguin with insufficient energy reserves at the beginning of moult has a reduced chance of survival (Green et al., 2004; Whitehead et al., 2017). Therefore, the moult season usually coincides with periods of high prey availability so that enough energy reserves can be accumulated following the breeding season and prior to moult (Wolfaardt et al., 2009; Sherley et al., 2014).

Factors that drive the timing and duration of moult in birds have previously been investigated (Pageau et al., 2020; Zuberogoitia et al., 2018). Photoperiod, or day length, is one extrinsic factor known to influence the timing of moult as well as breeding and migration (Bhardwaj et al., 2013; Dixit and Sougrakpam, 2013), while body condition is an intrinsic factor known to influence the timing of moult events for some bird species (Remisiewicz et al., 2010; Salton et al., 2015). Importantly, the onset and duration of moult appears to be flexible, with some species capable of delaying onset, usually to attempt another round of breeding, after which they can increase the rate of feather regeneration (Dawson, 2004; Morrison et al., 2015). Such plasticity is believed to be important for individuals faced with decreasing food resources during periods of unfavourable weather conditions (Wolfaardt et al., 2009). Despite the advantages of delaying moult for an additional breeding attempt, accelerating the duration of moult has negative impacts on the quality of the plumage (Dawson, 2004).

The little penguin (*Eudyptula minor*), the smallest of all penguin species, breed along the southern coasts of Australia and New Zealand, with much of the population on Bass Strait Islands (Arnould et al., 2004; Ganendran et al., 2016). Anthropogenic changes in sea surface temperatures (SST) are expected to impact prey abundance, affecting little penguin foraging, breeding, and survival, particularly if sufficient energy reserves cannot be accrued prior to or after moult (Green et al., 2004; Whitehead et al., 2017; Spillman et al., 2021; Berlincourt and Arnould, 2015; Jenouvrier et al., 2014; Lima and Estay, 2013). As little is known about the timing and duration of moult in little penguins, any impact that climate change could have in the lead up to, during, and following moult is currently speculative. To effectively predict the impacts of climate change on little penguins, a deeper understanding of moult and factors influencing the process is needed (Cullen et al., 2009). Therefore, the aims of this study were to: (1) identify the presence of inter-annual variation in the timing and duration of moult in little penguins; (2) identify important factors influencing these parameters and; (3) investigate which parameters influence the rate of body mass loss during moult.

## RESULTS

Over 2 years, data for 206 individuals were collected (90 in 2015 and 116 in 2016). The 2015 moult season was captured in full, with the last recording taken on 16 April. In 2016, however, the moult season was only partially observed, with fieldwork concluding on 19 March due to limitations imposed by the projects predefined timeline. As a result, fewer penguins were observed completing moult in 2016 and the number of individuals analysed varies throughout these results. All individuals were used to determine the timing of moult (i.e. the date that moult started, *n*=206, 90 in 2015 and 116 in 2016); however, only individuals that completed moult could be used to determine the duration of moult and mass loss over moult (in both cases, *n*=106, 84 in 2015 and 22 in 2016). These results are presented with the understanding that, due to the incomplete capture of the moult period in 2016, they underrepresent penguins that started moult later.

### The timing of moult

**Fig. 1.**
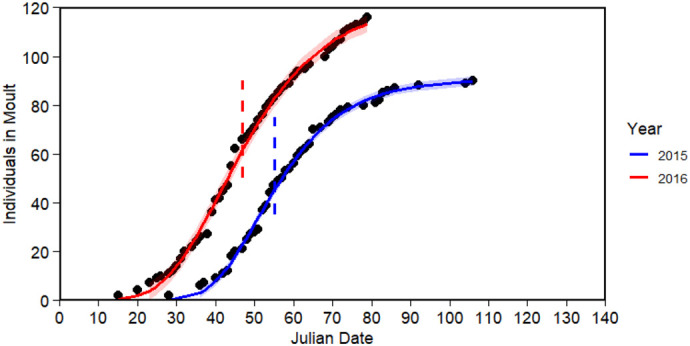
**The cumulative number of individuals arriving at nest boxes to commence moult in 2015 (blue;** ***n*****=90, 43.69% of all individuals) and 2016 (red,** ***n*****=116, 56.31% of all individuals).** Fitted Gompertz curves (solid blue and red line) were used to determine the median Julian start date for moult, which represents the time at which the cumulative moult reaches 50% of its maximum value (vertical blue and red dashed line). The 95% confidence s.e.m. is shown for each curve in shading.

Individuals commenced moult from 28 January 2015 and from 15 January 2016. A preliminary analysis using a Gompertz function estimated the median start date at 55.2 or 24 February 2015 (*n*=90, 43.69% of all penguins) and earlier in 2016 at 46.8 or 15-16 February 2016 (*n*=116, 56.31% of all penguins), suggesting an unadjusted inter-annual difference of 9 days (Fig. 1). Within each year, the moult start date was not significantly different between sexes (*t*-test: *P*>0.05 in both cases).

To account for other variables, and to provide a better estimate of differences in moult start date between years, a multiple linear regression model was run and included starting mass, sex, and year on the square root-transformed moult start date (*n*=206). Year was the only influential parameter, with moult beginning earlier in 2016 than in 2015. Given the low delta AICc, model averaging was applied (Table S1) and moult start date was found to be significantly affected by year (est.=−0.7167±0.1529 adj. s.e.m, *P*<0.001), which, when back transformed, indicated that moult started 9.6 days earlier in 2016 (Table 1). Comparatively, starting mass (*P*=0.2000) and sex (male relative to female, *P*=0.9030) did not have an effect.

**Table 1.** Model-averaged analysis of factors affecting the moult start date in little penguins (*n*=206)

Coefficient	est.	s.e.m.	adj. s.e.m.	*z*	*P*
Intercept	7.0639	0.7503	0.7527	9.3850	<0.0001
Year (2016)	−0.7167	0.1520	0.1529	4.6870	<0.0001
Start mass (g)	0.0006	0.0005	0.0005	1.2820	0.2000
Sex (male)	0.0204	0.1661	0.1670	0.1220	0.9030

Results from model averaging of multiple linear regression testing the effects of year (2015, 2016, a factor), starting mass (g, numeric) and sex (M/F, a factor) on the square root-transformed moult start date (Julian, numeric).

### The duration of moult

Moult duration was identified for 106 individuals. In 2015, moult duration ranged between 14 and 25 days with median duration of 18.0 days (*n*=84). In 2016, moult duration ranged between 12 and 22 days with median duration of 17.5 days (*n*=22). No significant difference in moult duration was observed between 2015 and 2016 (Wilcox rank-sum test: W=1074, *P*=0.240, *r*=0.12). Additionally, no significant difference in moult duration was found between sexes within each year (2015: W=955.5, *P*=0.497, *r*=0.07; 2016: W=48.5, *P*=0.462, *r*=0.16).

A multiple linear regression model revealed that moult duration was not influenced by sex, but was influenced by starting mass, with larger mass associated with a longer moult duration, by moult start date, with later start dates leading to shorter moult durations, and by year, with moult duration in 2016 being shorter compared to 2015. Although starting mass was retained in many top models (Table S2), its effect on moult duration was not statistically significant when model-averaged (*P*=0.3787; Table 2), contrasting with the initial regression model. However, the model averaging supported that moult start date consistently impacted duration; each day delay in moult start was associated with a reduction in duration of 0.0421 days (approximately 58 min, ±0.0163 adj. s.e.m., *P*=0.0096). Year also had a marginal but not statistically significant effect, with moult duration in 2016 shorter by 1.2981 days (*P*=0.0769), while sex was not a significant predictor (males compared to females, *P*=0.6516).

**Table 2.** Model-averaged analysis of factors affecting the moult duration in little penguins (*n*=106)

Coefficient	est.	s.e.m.	adj. s.e.m.	*z*	*P*
Intercept	17.9482	3.4571	3.4766	5.1630	<0.0001
Start mass (g)	0.0019	0.0021	0.0021	0.8800	0.3787
Moult start date (Julian)	−0.0421	0.0161	0.0163	2.5880	0.0096
Year (2016)	−1.2981	0.7284	0.7339	1.7690	0.0769
Sex (male)	−0.2055	0.4524	0.4550	0.4520	0.6516

Results from model averaging of multiple linear regression testing the effects of starting mass (g, numeric), moult start date (Julian, numeric), year (2015, 2016, a factor), and sex (M/F, a factor) on moult duration (days, numeric).

### Starting mass and mass loss

Start and end of moult mass (g) were recorded for 106 individuals. In 2015 (*n*=84), the median and range of the starting mass was 1650 g (range: 1280, 2010), and the final mass was 915 g (700, 1130). In 2016 (*n*=22), the median starting mass was 1680 g (1440, 1950), and the final mass was 945 g (760, 1180). Significant differences in starting mass were observed between sexes in both years, with males entering moult at a greater mass than females (2015: W=356.5, *P*<0.001, *r*=0.51; 2016: W=5.5, *P*<0.001, *r*=0.77). When sexes were pooled, no significant difference was found between 2015 and 2016, suggesting that interannual variation may have masked interannual differences (W=898.5, *P*=0.846, *r*=0.02). However, the absence of statistical significance should not be interpreted as evidence of no difference, and we acknowledge that pooling sexes may obscure sex-specific patterns and increase variance.

**Fig. 2.**
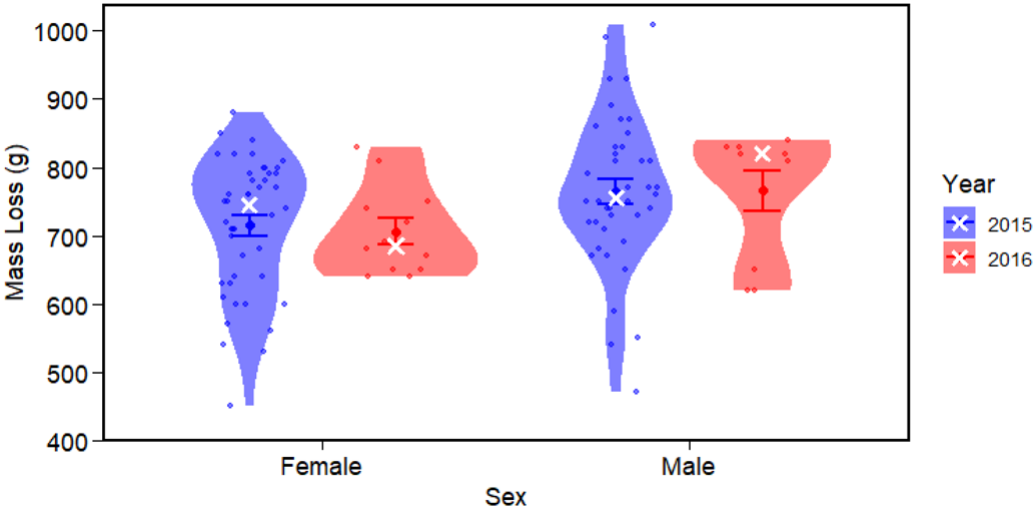
**Total body mass loss (g) during moult for female and male little penguins in 2015 (blue; F: 44, M: 40) and 2016 (red; F: 12, M: 10).** Violin plots illustrate the distribution of raw data points. Means±s.e.m. are overlaid in blue and red, while medians are indicated by a white ‘X’.

The median total mass loss was 750.0 g (450, 1010) in 2015 and 730.0 g (620, 840) in 2016, with a proportional mass loss of 0.45 (0.29, 0.58) and 0.45 (0.34, 0.52), respectively. The daily median mass loss rate was 49.0 g d^−1^ (31.2, 76.4) in 2015 and 50.3 g d^−1^ (38.0, 93.4) in 2016. A two-sample *t*-test revealed no significant difference in mass loss over moult between 2015 and 2016 (*P*=0.837). However, mass loss during moult was significantly different between sexes (*P*=0.010), with males losing more mass on average (764.4 g) compared to females (713.0 g) (Fig. 2). In 2015, individuals were measured repeatedly throughout moult, indicating that mass loss occurred at a relatively constant, linear rate. As equivalent repeated measurements were not obtained in 2016, these 2015 data are presented for reference only (Fig. S1) .

A generalised linear regression model including the effects of starting mass, sex, year, moult start date, and duration of moult on total mass loss identified moult duration and starting mass as key variables influencing mass loss (AIC in Table S3), with model-averaged coefficients supporting the results of the regression, showing statistical significance of moult duration and starting mass but limited biological relevance (Table 3). As a log link was used in the Gamma GLM, the estimated coefficients represent multiplicative effects or proportional change on mass loss. For each additional day of moult, mass loss increased by 0.0255 g (±0.0042 adj. s.e.m., *P*<0.001), which corresponds to an approximate 2.6% increase in mass loss per day. Additionally, for each additional gram of starting mass, total mass loss increased by 0.0006 g (±0.0001 adj. s.e.m., *P*<0.001), corresponding to about a 0.06% increase in mass loss per gram. Moult start date (*P*=0.6000), year (2016 compared to 2015, *P*=0.6910) and sex (male compared to female; *P*=0.8190) had no influence on mass loss.

**Table 3.** Model-averaged analysis of factors affecting the total moult mass loss (g) in little penguins (*n*=106)

Coefficient	est.	s.e.m.	adj. s.e.m.	*z*	*P*
Intercept	5.1660	0.1413	0.1429	36.1590	<0.0001
Moult duration (days)	0.0255	0.0041	0.0042	6.0890	<0.0001
Start mass (g)	0.0006	0.0001	0.0001	7.0970	<0.0001
Moult start date (Julian)	−0.0105	0.0199	0.0201	0.5250	0.6000
Year (2016)	0.0002	0.0004	0.0005	0.3970	0.6910
Sex (male)	0.0033	0.0141	0.0142	0.2290	0.8190

Results from model averaging of generalized linear model, testing the effects of moult duration (days, numeric), starting mass (g), sex (M/F), start date (Julian, numeric) and year (2015, 2016) on total moult mass loss (g, numeric).

## DISCUSSION

This study identified that moult characteristics were broadly consistent across the 2 years, with minimal interannual differences, though subtle shifts in timing and sex-specific patterns suggest biologically relevant trends warranting further investigation. A preliminary analysis estimated moult started about 9 days earlier in 2016 than 2015, refined to 9.6 days using model-averaged, back-transformed regression coefficients. The year was the only significant predictor of moult start date, though truncated 2016 data likely underrepresented later starters. Later moult onset was associated with shorter duration, with each day's delay reducing duration by approximately 58 min. Univariate comparisons showed males lost more mass than females, but in multivariate models only starting mass and moult duration predicted total mass loss. Despite statistical significance, effect sizes related to total mass loss were small, suggesting limited biological relevance from just 2 years’ data.

### Factors influencing the timing of moult

Timing for the moult start date in little penguins was affected by year, with moult commencing earlier in 2016 than in 2015, and not affected by sex or starting mass; we acknowledge, however, that data collection in 2016 was cut short which may influence this result. The Gompertz model indicated a 9-day difference between the median start date in each year, but the regression model, which adjusted for sex and starting mass, found a difference of 9.6 days once back transformed. This slight change from the cruder estimate suggests a potential effect of sex and starting mass, though the limited data may not have been sufficient to fully capture these effects. In any case, the relatively consistent start dates between years align with previous research on little penguins at Phillip Island, which also reported relatively stable start dates across years (Reilly and Cullen, 1983). While no definitive conclusion can be drawn regarding the role of photoperiod, a relatively fixed factor known to affect the onset and timing of moult (Dixit and Sougrakpam, 2013), the relatively small difference in start dates between years in this study suggest that moult timing may be affected by photoperiod, though, additional data would be required to further explore this effect in little penguins at Phillip Island.

Given the absence of a clear effect of sex and starting mass, but the presence of year-to-year variation, extrinsic factors such as local food availability and the need to reach a critical body mass to initiate moult may be key factors influencing moult timing. In this study, moult onset peaked in February, coinciding with the abundance of key prey species, pilchards (*Sardinops sagax*) and Australian anchovies (*Engraulis australis*), which are typically most plentiful during this period (Hobday, 1991). The effect of food availability on moult phenology is well-documented in bird species. For example, research on king penguins (*Aptenodyptes patagonicus*, Gauthier-Clerc et al., 2002) and African penguins (*Spheniscus demersus*, Sherley et al. 2014) illustrates how the timing of moult coincided with the greatest availability of prey and, therefore, body condition. By having prey availability constrain the timing of moult, the risk of starvation during moult is reduced (Enstipp et al., 2019; Whitehead et al., 2017). Therefore, like other Spheniscidae species, little penguins may rely on prey availability, and thus body condition, as the proximate cue to initiate moult, but further data is needed to accurately assess this.

Climate change and rising SST are predicted to impact the south-eastern coast of Australia with changes to oceanographic conditions expected to cause shifts in the foraging and breeding phenology of many seabird species (Berlincourt and Arnould, 2015; Chambers et al., 2014; Green et al., 2023). In little penguins at Penguin Island, Western Australia, warmer SST prior to the breeding season are thought to be linked to a decrease in breeding success due to a reduction in the abundance of local prey (Cannell et al., 2012). Not unlike the timing of the breeding season, changes to SST may negatively impact survivorship of little penguins during moult. As warmer SST may cause a reduction in local prey availability, little penguins that cannot delay the onset of moult any further and have failed to accumulate sufficient energy reserves prior to moult have a reduced chance of survival during the moult fast (Enstipp et al., 2019). However, for little penguins at Phillip Island, warmer SST during the late austral summer/early autumn have been correlated with earlier and longer breeding seasons and an increase in breeding success (Cullen et al., 2009). Despite this apparent positive effect of warmer SST on the breeding success of little penguins, earlier and longer breeding seasons often involve additional clutches being attempted (Berlincourt and Arnould, 2015). A longer breeding season may overlap with the moult start date (Chambers et al., 2014), and since moult cannot be prematurely ended, this overlap could increase the likelihood of chick abandonment; this has been observed in African penguins (*S. demersus*) at Western Cape, where some chicks were abandoned as parents could not delay the initiation of moult further (Sherley et al., 2014).

Local climate variables such as ambient air temperature, humidity and rainfall are also known to influence the survival of little penguins during moult. For example, low humidity and high rainfall during the moult period have resulted in high mortality rates of little penguins at Phillip Island (Ganendran et al., 2016). High humidity is thought to alleviate water loss and prevent dehydration during moult, whereas high rainfall and wet conditions make penguins more prone to hypothermia as their plumage is not fully insulative (Ganendran et al., 2016; Williams et al., 2015). As birds are unable to go to sea during the moult period, climate conditions have a greater influence on penguin survival during moult than the breeding period when birds can abandon breeding if conditions on land become unfavourable (Chambers et al., 2014). Therefore, measuring the extent to which environmental variables influence the timing of moult in little penguins is imperative in predicting the potential implications of climate change.

### Factors influencing the duration of moult

Moult start date and year were the only factors found to influence moult duration. An earlier moult start date was associated with a longer moult duration, with each additional day later reducing the duration by approximately 0.042 days (or ∼1 h). Year also had a marginal effect, with moult duration being 1.45 days shorter in 2016. For little penguins, moult usually starts during late summer when prey availability is still high (Ganendran et al., 2016). In contrast, prey availability is lowest during the winter foraging period following moult, resulting in an increase in mortality for many seabird species (Chambers et al., 2014; McCutcheon et al., 2011; Dann et al., 1991). For little penguins, the risk of mortality during the winter period is exacerbated if individuals finish moult with a low body mass (McCutcheon et al., 2011). The effect of moult start date on moult duration suggests that moult duration is influenced by the availability of food resources, as individuals beginning moult towards the end of the season must accelerate their moult duration to prepare for unfavourable conditions during winter (Rogers et al., 2014; Danner et al., 2015). In contrast, penguins that enter moult earlier when prey availability is still high do not need to accelerate their moult more than necessary. By exhibiting this degree of flexibility in response to periods of high fluctuating prey availability, birds can ensure that their body mass is regained following moult prior to periods of low prey availability (Carpenter-Kling et al., 2022; Danner et al., 2015). However, it has been shown that increasing the rate or duration of moult can compromise plumage quality as hastened feathers represent less overall quality and mass (Dawson, 2004). A hastened and therefore poorer quality plumage has been linked to a decreased chance of survival, as insulation against ocean temperatures and waterproofing, required for efficient swimming and therefore foraging, are compromised (Dawson, 2004). Birds can also exhibit a delayed and therefore accelerated moult to extend the breeding season (Flinks et al., 2008; Rogers et al., 2014). Therefore, the rapid completion of moult in response to a delayed or late start is observed as being a direct trade-off between increasing short-term breeding success or the optimal maintenance of the integument (Flinks et al., 2008). However, whether little penguins exhibit this penalty when the moult process is accelerated is unknown, as long-term data is needed to assess previous breeding success and subsequent mortality.

### Mass loss during moult

In this study, we calculated that penguins lost approximately 45% of their body mass over the course of moult lasting 17.5 to 18 days, equating to ∼49.0 g d^−1^. We acknowledge that, with only start and end mass values, the assumption of linear mass loss is inherent, as two points necessarily define a straight line. However, previous studies on penguin species [e.g. Green et al. (2004)] indicate that mass loss during moult typically occurs at a relatively steady rate, supporting the use of a linear approximation in this context. Furthermore, data collected in only 2015, when repeated mass measurements were taken from the same individuals throughout the moult period, support a linear pattern of mass loss (see Fig. S1). Consistent with this, a model-averaged analysis from both years revealed significant positive relationships between total mass loss and both moult duration and starting mass, while year, sex, and moult start date were not significant predictors. These findings further support the suitability of a linear fit for modelling mass loss across the moult period. Linear mass loss, also known as Phase II mass loss, is indicative of the primary use of endogenous lipid reserves (Cherel et al., 2005; Verrier et al., 2012). As lipids have a higher caloric density than protein, penguins may catabolize endogenous lipids to increase the efficiency of the moult fast (Cherel et al., 1994; Enstipp et al., 2019), and by doing so, can decrease the risk of starvation during moult (Green et al., 2004). In king penguins (*A. patagonicus*), endogenous lipids are sourced from the integument and provide 61% of the energy required to manage increased thermoregulatory demands (Cherel et al., 1994). In contrast, endogenous proteins are the primary energy reserves used in both Phase I and Phase III mass loss, states of high daily body mass loss; endogenous proteins are also used to provide amino acids required for feather synthesis (Portugal and Guillemette, 2011). Within macaroni penguins (*E. chrysolophus*), Phase II mass loss was the only fasting phase observed while penguins were ashore, and both Phase I and Phase III occurred whilst penguins were at sea (Green et al., 2004). Therefore, it is likely that little penguins in this study are exhibiting a similar strategy to macaroni penguins as Phase II was predominately observed within this study.

The daily rate of mass loss was influenced by both moult duration and start-of-moult mass, with longer moult duration and higher initial body mass associated with greater mass loss. These findings suggest that mass loss in little penguins reflects the length of their fasting period and the stored energy reserves available to them that act to reduce the risk of starvation (Enstipp et al., 2019). As penguins cannot forage during moult, sufficient fat reserves are essential to meet basal metabolic needs, including thermoregulation. Additional energy demands arise during moult due to increased thermoregulatory costs (Enstipp et al., 2019) and the use of protein for feather regeneration (Portugal and Guillemette, 2011). Consequently, moult is a period of increased mortality for penguins (McCutcheon et al., 2011; Dann et al., 1991), and individuals entering moult at low body mass may not survive the process. Further, penguins undergoing a rapid moult may experience greater daily mass loss, potentially due to the energetic cost of accelerating feather replacement, but this would facilitate the completion of moult whilst prey availability is still relatively high (Dawson, 2004). In contrast, penguins that enter moult at a high starting mass are less likely to starve during moult and, therefore, can afford to use more energy reserves per day. Considering the costs of fasting while in moult (increased thermoregulatory demands and feather synthesis), it is likely that little penguins are regulating their rate of body mass loss (metabolic downregulation and efficient use of fat stores) in response to a long duration and/or a low starting mass, to avoid starvation (Green et al., 2004; Baudinette et al., 1986). Exhibiting flexibility in the amount and type of energy reserves used benefits animals by minimizing the risk of completing moult at a critical mass loss when mortality is highly probable (Fox and Kahlert, 2005).

## MATERIALS AND METHODS

### Study site and data collection

**Fig. 3.**
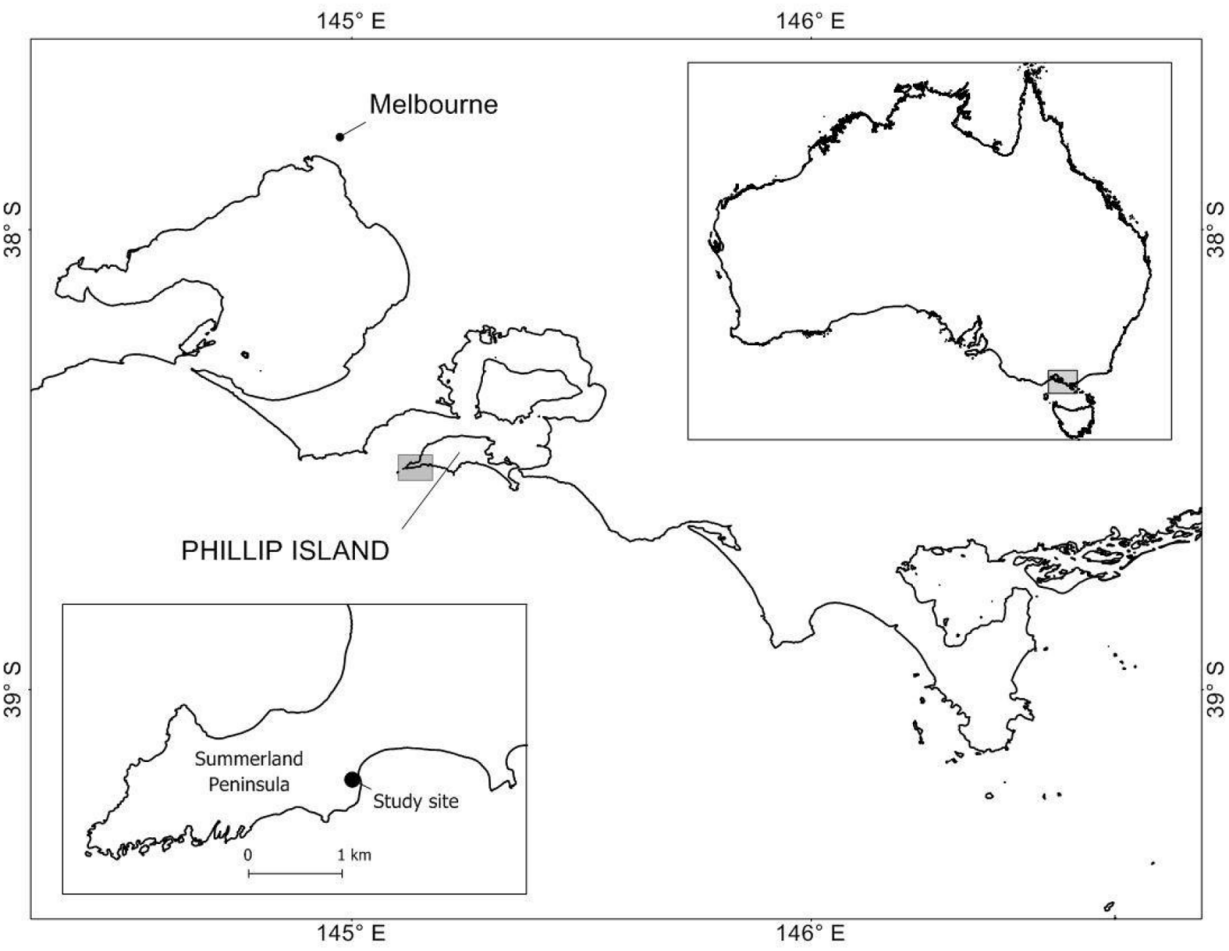
**Study site within the Summerland Peninsula on the western end of Phillip Island, Victoria.** The study site is represented by ●.

Fieldwork was conducted on the Summerland Peninsula (Fig. 3) at Phillip Island, in south-eastern Australia (38°30′S, 145°10′E), under Deakin University Animal Ethics Committee under permit number AEX12-2015 and in accordance with the Phillip Island Nature Parks Ethics Committee under permit number 32014. This regenerated area consists of grasslands, woodlands, succulent vegetation and hundreds of artificial nest boxes that have been installed to promote breeding activities for little penguins (Sutherland and Dann, 2014). For this study, 125 of the most undisturbed and accessible nest boxes were selected.

**Fig. 4.**
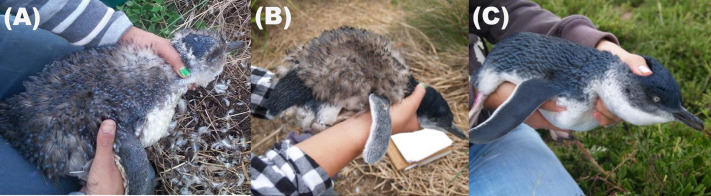
Catastrophic moult in little penguins is characterized by the expulsion of old feathers by (A) new feathers from within the epidermis, (B) a successive renewal of feathers and (C) finally a complete, renewed plumage.

Beginning in mid-January of 2015 and 2016, the nest boxes were visited daily and checked along with surrounding vegetation for penguins. Penguins were examined and their moult status (Fig. 4) was determined using previously described keys (Reilly and Cullen, 1983). Once an individual was considered to have commenced moult, morphometric measurements [body mass (g), bill depth (mm)] were taken using a cloth bag to hold the individual, a spring balance (±10 g) for mass and Vernier callipers (±0.1 mm) for bill measurements. Sex was determined from bill depth using a discriminate function (Arnould et al., 2004). If individuals were not identifiable from previously attached Australian Bird and Bat Banding Schemes identification tags, a unique identifier transponder microchip (TIRIS™) was injected subcutaneously in the dorsal region (Dann et al., 2014). As many penguins lacked an existing identifier, their age could not be determined, despite age being a known factor influencing moult in other species (Kemper et al., 2008). Penguins were then returned to the nest box. In 2015, penguins were visually examined and weighed every five days to monitor their moult process. If any individuals were not located during the scheduled checks, additional searches were conducted on the following days. In 2016, only visual examinations were conducted every 5 days to minimise disturbance.

The moult start date was defined as the first day that an individual was observed in moult while ashore. The duration of moult (days) was calculated from the moult start date to the last sighting of an individual with fully renewed plumage. As penguins remain ashore throughout moult and return to sea upon completion to commence foraging (Gales and Green, 1990), this ashore period is a good representation of moult duration. The starting and final mass (g) were recorded at the first and last capture of an individual. Total mass loss (g) was calculated by subtracting the final mass from starting mass, and average mass loss per day (g d^−1^) was calculated by dividing the total mass loss by the duration of moult (days). Proportional mass loss was calculated by subtracting the final mass from starting mass and dividing by starting mass.

### Data processing and statistical analysis

All statistical analyses were conducted using the R Statistical software interfaced with RStudio (version 4.4.0) (R Core Team, 2023). Data are presented as means±standard error (s.e.m.) and unless otherwise stated (see below), *P*<0.05 is significant. For all data, Shapiro–Wilk tests were used to assess normality and, when not normal, the data underwent secondary tests (see below) and received the appropriate transformation (Feng et al., 2014; Liang et al., 2009).

To gain a crude estimate of the moult start date for each year, a nonlinear least squares analysis, in which the experimental points of the moult start date accumulation curve are fitted to the Gompertz function, was used. This approach was employed to examine the moult start date for each year and provide an initial estimate of the median moult start date, given that the data was skewed (Bates and Chambers, 1992; y Galán et al., 2015). Each median moult start date was estimated by determining the time at which the cumulative moult reaches 50% of its maximum value. Independent two-sample *t*-tests were used to compare moult start dates between sexes within each year, after confirming normality with Shapiro–Wilk tests. For an estimate that better captured the difference in moult start date between years, and accounted for variables affecting start date, a multiple linear regression model was used. This multiple linear regression model examined the effects of sex (M/F, a factor), year (2015, 2016, a factor), and starting mass (g, numeric) on moult start date (Julian, numeric), which was square root-transformed to reduce skewness and improve normality of residuals. To quantify the effect of the year in days, the model-averaged coefficients were used to calculate predicted values. First, the predicted moult onset on the transformed scale for the reference year (2015) was taken from the model intercept, while the value for 2016 was calculated by summing the intercept and the coefficient for the year (2016) term. These two values were then back transformed into days by squaring them. The final effect size was determined by calculating the difference between these back-transformed predictions.

Moult duration (days) was examined using a Wilcoxon rank-sum test to compare moult duration (days) between the years (2015 and 2016), due to non-normality (Myles et al., 1973) and an effect size was calculated to test if there was a relationship despite the sample size (Package *rstatix*, *wilcox_effsize*). As there were more data in 2015 (*n*=84) than 2016 (*n=*22), data were split, and a Wilcoxon rank-sum test was used to assess whether within years the moult duration was affected by sex (M/F); an effect size was also calculated. A multiple linear regression model was used to examine the effects of sex (M/F, a factor), year (2015, 2016, a factor), starting mass (g, numeric) and start date (Julian, numeric) on moult duration (days, numeric). Although moult duration was not normally distributed, visual inspection (histogram, Q-Q plot) and Shapiro–Wilk tests of the regression residuals confirmed approximate normality.

Starting mass (g) was examined with Wilcox rank-sum tests to determine differences between years (2015, 2016) for each sex (M/F), and between years when pooling sex. Rate of mass loss was calculated by dividing final mass by the duration of moult. Independent two-sample *t*-tests were conducted to examine differences in mass loss during moult by year and sex, after examining normality with Shapiro–Wilk tests. To model mass loss during moult, a generalized linear model (GLM) with a Gamma distribution and a log link was used due to the positively skewed data. Predictor variables included sex (M/F, a factor), initial body mass (g, numeric), moult duration (days, numeric), start date (Julian, numeric), and year (2015, 2016, a factor). Model assumptions were assessed using Q-Q plots and residual-versus-fitted plots. Residuals were not normally distributed, which is acceptable in Gamma GLMs where normality of residuals is not required. As a log link was used, estimated coefficients represent multiplicative effect or proportional change on mass loss.

For each model, the influence of explanatory variables on the response was assessed using the dredge function within the *MuMIn* package (Barton and Barton, 2015). Visual inspection of the residuals was conducted to assess whether models followed an approximately normal and unbiased distribution. Sub-models were compared using the Akaike information criteria (AIC), corrected for small sample size. The delta AIC and Akaike weights were used to evaluate model support, with the model with the lowest AIC considered to be the best supported (Brunham and Anderson, 2002; Symonds and Moussalli, 2011). The AIC tables are presented in supplementary materials. The 95% confidence intervals were examined, and a parameter was influential if its confidence interval did not pass through zero (Arnold, 2010). Where appropriate, further model averaging was achieved using *model.avg* from the *MuMIn* package (Barton and Barton, 2015). This function combines coefficients from supported candidate models, weighing each model's contribution by its AICc weight. Such an approach provides more robust parameter estimates by incorporating model selection uncertainty. In the main text, tables of the model-averaged coefficient estimates (est.), standard error (s.e.m.), adjusted standard error (adj. s.e.m.), *z*-value, and *P*-value for each predictor variable are presented; coefficients were calculated using the full model average, where the parameter estimate is set to zero for models in which the variable was not included.

## Supplementary Material

10.1242/biolopen.061989_sup1 Supplementary information
