# Supplementary material for: Mass loss, timing and duration of catastrophic moult in little penguins
Source: Biol Open. 2025 Sep 2;14(9):bio061989. doi: 10.1242/bio.061989 (PMC12444856; doi:10.1242/bio.061989)
Supplement: Supplementary information [file biolopen-14-061989-s1.pdf]

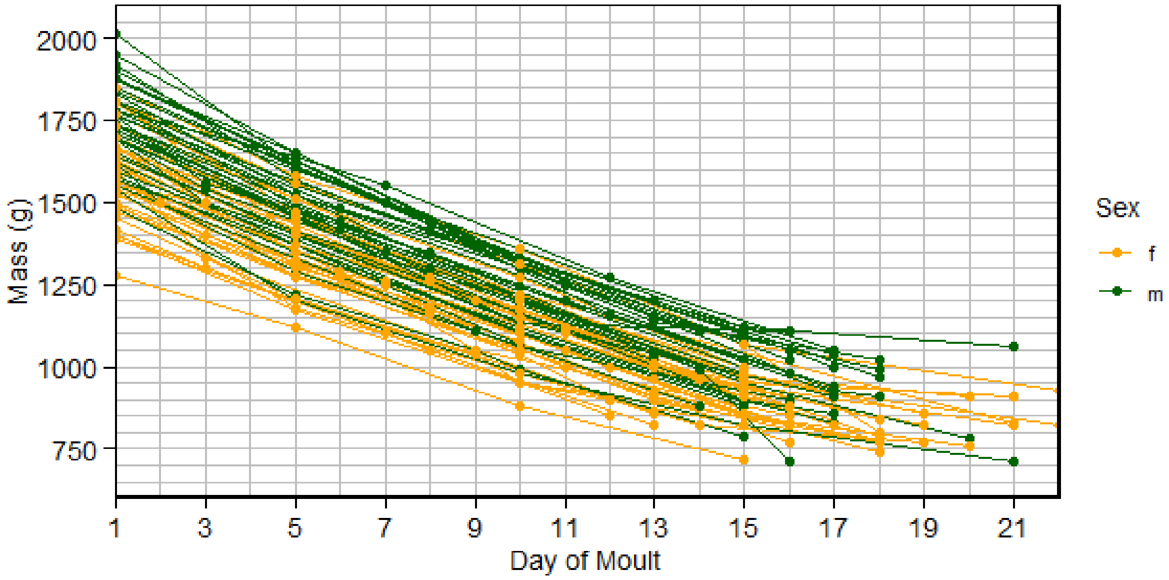

Fig. S1. Mass loss during moult in 2015 for 84 penguins; 44 females (orange), 40 males (green). Each line represents a single individual.

Table S1. Akaike's Information Criterion based model selection of multiple regressions models for moult start date (Julian) in little penguins (*n* = 206), including starting mass (SM), sex (Sx) and year (Yr). The model-averaged factors affecting moult start date are presented in **Table 1**.

Estimate	SM	Sx	Yr	DF	logLik	AICc	ωi
7.5			+	3	-304.9	615.9	0.40
6.5	0.0006		+	4	-304.0	616.2	0.33
7.5		+	+	4	-304.8	617.8	0.15
6.4	0.0007	+	+	5	-304.0	618.3	0.12

Table S2. Akaike's Information Criterion based model selection of multiple regressions models for moult duration (days) in little penguins from a multiple linear regression model (*n* = 106), including starting mass (SM), sex (Sx), year (Yr) and moult start date (MSD). The model-averaged factors affecting moult duration are presented in **Table 2**.

Estimate	SM	Sx	Yr	MSD	DF	logLik	AICc	ωi
17.0	0.0025		+	-0.0436	5	-238.0	486.6	0.25
14.9	0.0040	+	+	-0.0453	6	-237.0	486.9	0.22
21.0			+	-0.0425	4	-239.3	486.9	0.22
21.1		+	+	-0.0426	5	-239.2	489.1	0.08
20.0				-0.0283	3	-241.9	490.1	0.05
16.1	0.0023			-0.0290	4	-240.9	490.2	0.04
14.3	0.0037	+		-0.0299	5	-240.2	490.9	0.03
18.4					2	-243.8	491.7	0.02
14.7	0.0022				3	-242.9	492.0	0.02
18.6			+		3	-243.0	492.2	0.02
20.0		+		-0.0283	4	-241.9	492.2	0.02
14.8	0.0023		+		4	-242.0	492.4	0.01
13.0	0.0034	+			4	-242.3	493.1	0.01
13.0	0.0035	+	+		5	-241.4	493.4	0.01
18.4		+			3	-243.8	493.8	0.01
18.6		+	+		4	-243.0	494.3	0.01

Table S3. Akaike's Information Criterion based model selection of generalized linear models for total mass loss (g) in little penguins (*n* = 106), including starting mass (SM), sex (Sx), year (Yr), moult start date (MSD), and mount duration (MD). The model-averaged factors affecting total moult mass loss are presented in **Table 3**.

Estimate	SM	Sx	Yr	MSD	MD	DF	logLik	AICc	ωi
5.2	0.0006				0.0253	4	-604.3	1217.0	0.29
5.1	0.0006	+			0.0249	5	-603.6	1217.8	0.20
5.2	0.0006			0.0005	0.0260	5	-603.9	1218.5	0.14
5.2	0.0006		+		0.0256	5	-604.2	1219.1	0.10
5.1	0.0006	+		0.0005	0.0255	6	-603.3	1219.4	0.09
5.1	0.0006	+	+		0.0251	6	-603.5	1219.9	0.07
5.1	0.0005		+	0.0008	0.0269	6	-603.5	1219.9	0.07
5.1	0.0006	+	+	0.0007	0.0263	7	-603.0	1221.1	0.04

Biology Open • Supplementary information
